# Function analysis of *GhWRKY53* regulating cotton resistance to verticillium wilt by JA and SA signaling pathways

**DOI:** 10.3389/fpls.2023.1203695

**Published:** 2023-06-02

**Authors:** Youzhong Li, Haihong Chen, Youwu Wang, Jincheng Zhu, Xiaoli Zhang, Jie Sun, Feng Liu, Yiying Zhao

**Affiliations:** ^1^ Key Laboratory of Oasis Eco-Agriculture, College of Agriculture, Shihezi University, Shihezi, China; ^2^ Xinjiang Production and Construction Group Key Laboratory of Crop Germplasm Enhancement and Gene Resources Utilization, Cotton Research Institute, Xinjiang Academy of Agricultural and Reclamation Science, Shihezi, China; ^3^ College of Plant Science and Technology, Tarim University, Alar, China

**Keywords:** *Gossypium hirsutum*, verticillium wilt, virus virus-induced gene silencing, *GhWRKY53*, jasmonic acid, salicylic acid

## Abstract

WRKY transcription factors (TFs) play an important role in regulating the mechanism of plant self-defense. However, the function of most WRKY TFs in upland cotton (Gossypium hirsutum) is still unknown. Hence, studying the molecular mechanism of WRKY TFs in the resistance of cotton to Verticillium dahliae is of great significance to enhancing cotton disease resistance and improving its fiber quality. In this study, Bioinformatics has been used to characterize the cotton WRKY53 gene family. we analyzed the GhWRKY53 expression patterns in different resistant upland cotton cultivars treated with salicylic acid (SA) and methyl jasmonate (MeJA). Additionally, GhWRKY53 was silenced using a virus-induced gene silencing (VIGS) to determine the contribution of GhWRKY53 to V. dahliae resistance in cotton. The result showed that GhWRKY53 mediated SA and MeJA signal transduction pathways. After VIGS of the GhWRKY53, the ability of cotton to resist V. dahliae decreased, indicating that the GhWRKY53 could be involved in the disease resistance mechanism of cotton. Studies on the levels of SA and jasmonic acid (JA) and their related pathway genes demonstrated that the silencing of GhWRKY53 inhibited the SA pathway and activated the JA pathway, thereby reducing the resistance of plants to V. dahliae. In conclusion, GhWRKY53 could change the tolerance of upland cotton to V. dahliae by regulating the expression of SA and JA pathway-related genes. However, the interaction mechanism between JA and SA signaling pathways in cotton in response to V. dahliae requires further study.

## 1 Introduction

Upland cotton is an important economic crop in China and one of the major cultivars grown in the northwest of the country due to its high yield and good fiber quality. In recent years, *Verticillium dahliae* outbreaks were frequent in cotton due to changing climatic conditions, long-term monocultures, and the frequent introduction of new cotton varieties across the globe (Shaban et al., 2018). *V. dahliae* is a soil-borne, semi-living parasitic plant pathogenic fungus that causes verticillium wilt in cotton. It has the characteristics of wide distribution, harmful effects, strong infectivity, and is very hard to cure (Klosterman et al., 2009). Due to the absence of a targeted control agent for infected plants (Fradin et al., 2006), *V. dahliae* seriously affects cotton yield in China.

Recently, it was established that secondary metabolites and hormones were involved in cotton disease resistance. When plants sense signals of pathogenic fungus invasion, self-defense responses are regulated through hormonal signal transduction. Currently, salicylic acid (SA), jasmonic acid (JA), ethylene (ET), abscisic acid (ABA), gibberellins (GAs), and brassinosteroids (BRs) are widely studied. In *Gossypium barbadense* disease-resistant cultivars inoculated with *V. dahliae*, it was found that the content of SA increased, and the expression of SA synthesis genes (PAL, phenylalanine ammonia-lyase and ICS, Isochorismate synthase) and downstream reaction genes (NPR1, nonexpressor of pathogenesis-related genes 1 and PR1, polyadenylic acid 1) also increased (Zhang et al., 2013; Zhang et al., 2017). The exogenous application of methyl jasmonate (MeJA) to cotton activated the expression of PR genes in the jasmonate signaling pathway and enhanced plant disease resistance (Li et al., 2014). Similar results were also obtained after spraying with ET (Guo et al., 2016).

As a plant-specific class of transcriptional regulators, WRKY transcription factors (TFs) possess conserved domains that determine their functions and provide a basis for the classification of the WRKY family of TFs (Eulgem et al., 2000). Domain C of WRKY TFs has a characteristic zinc finger structure with seven amino acid residues of conserved WRKYGQK in the N-terminus. This helps in its specific binding to w-box (T/C) TGAC (T/C) sites in genes regulating phytohormone signaling, including ABA, ET, JA, and SA, and in the disease resistance mechanisms of cotton (Pandey and Somssich, 2009; Zhang et al., 2015). Studies showed that WRKY TFs specifically recognize w-box sites in the promoters of the *PR* of SA signaling pathways to promote disease resistance response (Rushton and Somssich, 1998). WRKY TFs, including those in Capsicum *CaWRKYd* (Huh et al., 2012), tobacco *NtWRKY12* (Van et al., 2011), *Populus trichocarpa PtrWRKY73* (Duan et al., 2015), *OsWRKY53* (Zhang et al., 2015), *etc* have been shown to regulate plant resistance to pathogens by inducing the expression of the *PR* in the SA, ABA, and GA signaling pathways.

As a member of the *WRKY* family, the *WRKY53* transcription factor was first discovered in *Arabidopsis* leaves and was found to be specifically expressed in the early stage of leaf senescence (Hinderhofer and Zentgraf, 2001). Wan et al. (2004) through transgenic expression experiments on tobacco, it was demonstrated that active mutant *NtMEK2* induced the expression of *WRKY33* and *WRKY53*, thereby confirming that this class of transcription factors played a role in signal transmission. Murray et al. (2007) identified that *WRKY53* belonged to type III WRKY transcription factors and that most of the type III WRKY transcription factors were associated with plant resistance to pathogenic fungus. In addition, it had been reported that *WRKY53* could be a major factor affecting JA and SA synergy (Miao and Zentgraf, 2007; Shang et al., 2011). Luna and Ton (2012) found that plant immune response to pathogen invasion could be inherited under disease pressure. Also, after plant infestation, a transgenerational defense phenotype was developed through the activation of SA-induced defense genes, including *GENE1*, *WRKY 6*, and *WRKY53*.

Upland cotton, the largest cultivated variety in China, strongly supports the country’s national economic development. However, the invasion of *V. dahliae* has brought great loss to cotton farmers. With no chemical agents available against *V. dahliae*, cotton farmers resort to the use of cultivation measures, including stubble rotation, mid cultivation, and deep turning, to alleviate the effects of verticillium wilting. Apart from cultivation measures, the most economical and effective measures are to cultivate and promote disease-resistant varieties. Research on disease-resistant genes is of great significance to breeding disease-resistant cotton varieties. In this study, we identified the *GhWRKY53* and analyzed its expression patterns in different resistant upland cotton cultivars treated with SA and MeJA. *GhWRKY53* was silenced using VIGS to determine the contribution of *GhWRKY53* to *V. dahliae* resistance in cotton. Changes in the levels of SA and MeJA and their roles in the expression of *GhWRKY53* to *V. dahliae* resistance in cotton were also studied. Our study provides a theoretical basis for investigating the signaling pathways and molecular mechanisms of cotton resistance to *V. dahliae* and the breeding of high-resistance cultivars.

## 2 Materials and methods

### 2.1 Identification of *GhWRKY53* family genes

The genomes of *G. raimondii*, *G. arboreum*, *G. hirsutum* and *G. barbadense* were downloaded from CottonGen (https://www.cottongen.org/). Blastp (E-value ≤ 10^-10^) was carried out in cotton genome using *Gh*WRKY53 encoded amino acid sequence as a probe to obtain WRKY53 protein sequence in each of the four cotton species. Multiple sequence alignment of *GhWRKY53* was performed with DNAMAN (Kumar et al., 2016). Conserved motifs of the WRKY53 protein sequence were predicted with the use of online software, MEME (https://meme-suite.org/). RT-qPCR was used to analyze the gene expression level of *GhWRKY53* in the roots, stems, and leaves of resistant and susceptible cotton varieties.

### 2.2 Virus-induced gene silencing of *GhWRKY53*

Tobacco rattle virus (TRV) derived vectors, pTRV1 and pTRV2, were used for VIGS (Liu et al., 2002). The VIGS vector construction and experimental procedure were carried out according to the protocol previously described (Lu et al., 2021). The fragment targeting *GhWRKY53* was amplified using the forward primer of 5’-GAATTCGGGCAAAAAGACATCCTGGG-3’ and the reverse primer 5’-GGTACCGAAAGAAGTTGCCATCTCGGT-3’ (the underlined nucleotides in the forward and reverse primers represent the restriction sites of *Eco* R1 and *Kpn* I, respectively). The PCR cycles and confirmation of the fragment were done according to Lu et al. (2021). The cotton variety used in VIGS was Upland cotton ZZM2 and XLZ7. The method of *V. dahliae* infection of cotton was done according to Li et al. (2023). All primers used for the VIGS were designed with Primer3 software and are shown in **Table S1**.

### 2.3 Determination of endogenous SA and MEJA contents in cotton after *V. dahliae* infection

For the SA and MeJA treatment experiments, two-leaf stage seedlings of cotton were sprayed with 1mmolL^−1^ SA or 100 µmol L^−1^ MeJA (Xiong et al., 2019). Patients treated for 0 hours were selected as the control group. Collect root samples of cotton seedlings at 0, 6, 12, 24, and 48 hours after treatment. Roots of TRV::00 and TRV::*GhWRKY53* inoculated with *V. dahliae* for 0 and 48 h were selected to determine the endogenous levels of SA and MEJA. Moreover, the roots from five individual seedlings of cottons at two-leaf stage were collected to measure the expression profiles of *GhPAL*, *GhPR1*, *GhAOS1*, *GhPDF1.2* and *GhLOX* after *V.dahliae* infection in TRV::00 and TRV::*GhWRKY53* plants by qRT-PCR. The differences between groups were compared using Student’s *t*-test (* P < 0.05; ** P < 0.01).

### 2.4 qRT-PCR analysis

Total RNA was extracted from leaves of TRV1/TRV2::*00* or TRV1/TRV2::*GhWRKY53* treated ZZM2 and XLZ7 plants (both control and *V.dahliae* treated), and then reverse transcribed into cDNA to be used in qRT-PCR to analyze the effect of inoculation with *V.dahliae* treatment on gene expression changes. qRT-PCR was carried out by the SYBR Green (Roche, Rotkreuz, Switzerland) on a Light Cycler 480II (Roche, Germany) with default parameters. All primers used for the validation experiments were designed with Primer3 software and are shown in **Table S1**. The *GhUBQ7* gene served as an internal control to normalize differences between samples. The relative expression levels of genes from three biologically independent experiments were calculated using the 2^-ΔΔCT^method (Livak and Schmittgen, 2001).

### 2.5 Statistical analysis

SPSS 26.0 (SPSS, Chicago, USA) was used for data processing and analysis of variance. The data were analyzed by a one-way analysis of variance (ANOVA) and the significance of the difference was tested using the Duncan multiple comparison method. Origin 2022 (OriginLab, Northampton, USA) was used to plot the figures.

## 3 Results

### 3.1 Sequence alignment and phylogenetic analysis of *GhWRKY53*

**Figure 1:**
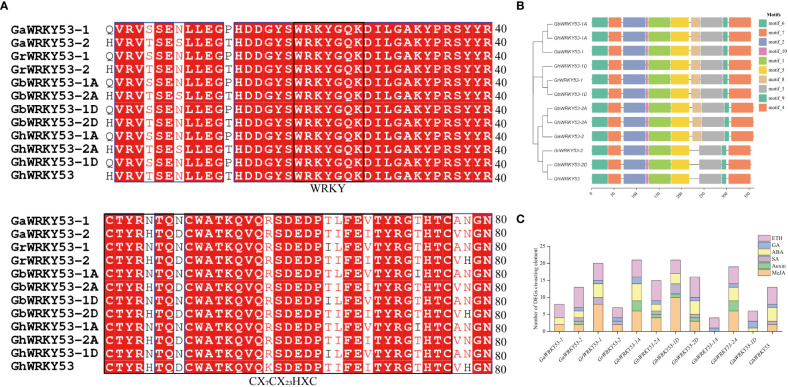
Sequence alignment, motifs, cis-acting elements analysis of *WRKY53.* **(A)** Multiple sequence alignment of cotton *WRKY53* gene. **(B)** Motif of 12 *WRKY53* proteins. Ten motifs were investigated using the MEME online tool. **(C)** Statistical analysis of the types and quantity of cis-acting elements in the promoter of WRKY53. *GaWRKY53-1 (Ga08G1308), GaWRKY53-2 (Ga12G0445), GrWRKY53-1 (Gorai.004G134600), GrWRKY53-2 (Gorai.008G253300), GbWRKY53-1A (GB_A08G1300), GbWRKY53-2A (GB_A12G2642), GbWRKY53-1D (GB_D08G1397), GbWRKY53-2D (GB_D12G2648), GhWRKY53-1A (GH_A08G1141), GhWRKY53-2A (GH_A12G2543), GhWRKY53-1D (GH_D08G1341), GhWRKY53 (GH_D12G2563)*.

The 2, 2, 4, and 4 *WRKY53* genes were identified in *G. arboreum*, *G. raimondii*, *G. barbadense*, and *G. hirsutum*, respectively. Further, the conserved domains of *GhWRKY53* were demonstrated by multiple protein sequence alignments of *GhWRKY53*. Almost all proteins contained the WRKY domain and zinc finger structure in the form of CX7C23HXC (**Figure 1A**). Based on the results of a motif analysis of *GhWRKY53* proteins using MEME suite, a total of ten conserved motifs were identified. *GrWRKY53-2*, *GbWRKY53-2D* and *GhWRKY53* contained nine motifs, while the others contained ten motifs (**Figure 1B**). Additionally, the promoter region of *WRKY53* in each cotton species contained at least two plant hormone *cis*-acting elements (**Figure 1C**). Of them, an ETH *cis*-responsive element was found in the promoter region of each *WRKY53* gene. And a MeJA *cis*-responsive element and an SA were found in four WRKY53 (*GaWRKY53-2*, *GbWRKY53-2A*, *GbWRKY53-1D* and *GbWRKY53-2D*). The results also showed that the *GaWRKY53-1*, *GhWRKY53-1A* and *GhWRKY53-1D* exclude MeJA and SA *cis*-responsive element (**Figure 1C**). The above results indicated that cotton *WRKY53* was regulated by a variety of hormones and was involved in different types of hormone response.

### 3.2 Analysis of *GhWRKY53* expression pattern

**Figure 2:**
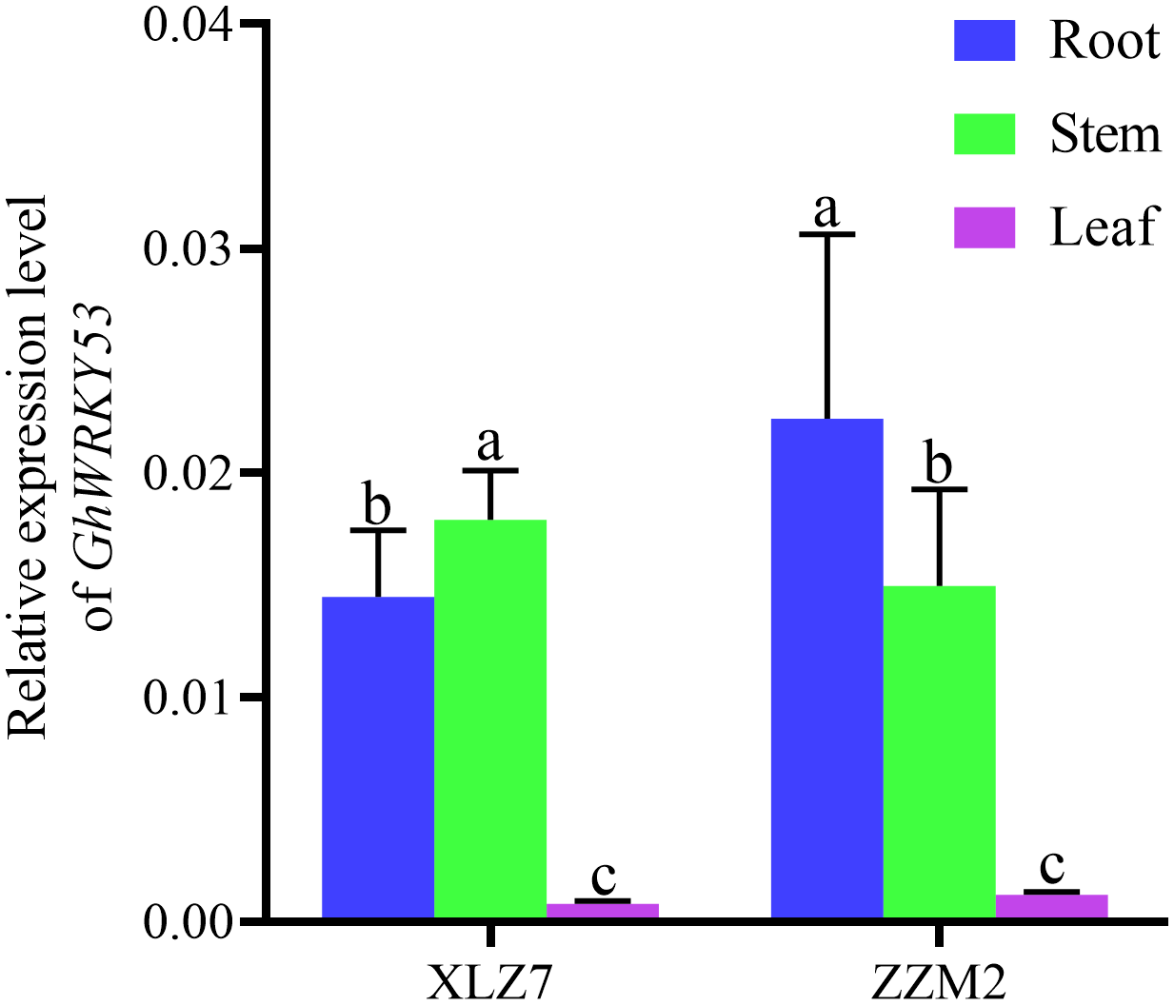
Expression levels of GhWRKY53 in cotton roots, stems and leaves. XLZ7: V. dahliae-susceptible cotton vatiety; ZZM2:V. dahliae-resistant cotton vatiety. GhUBQ7 is an internal reference gene. The data are three independent biological replicates. Different lowercase letters indicate significant differences (P < 0.05) between groups determined using Duncan’s multiple range test.

**Figure 3:**
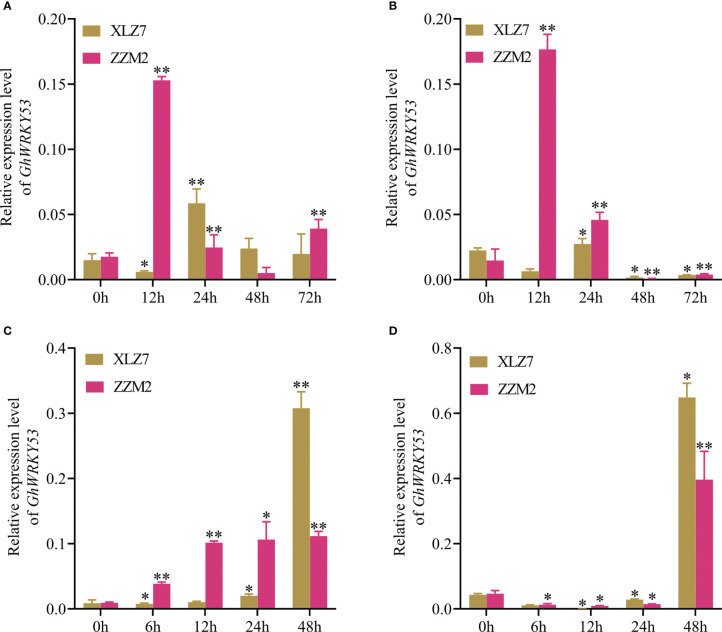
Expression levels of *GhWRKY53* after *V.dahliae* inoculation and SA, MeJA treatment. **(A)** The relative expression levels of *GhWRKY53* in cotton roots with *V.dahliae* inoculation at 0, 12, 24, 48 and 72 h. **(B)** The relative expression level of *GhWRKY53* in cotton stems with *V.dahliae* inoculation at 0, 12, 24, 48 and 72 h. **(C)** The relative expression levels of *GhWRKY53* in cotton roots with SA at 0, 12, 24, 48 and 72 h. **(D)** The relative expression level of *GhWRKY53* in cotton stems with MeJA at 0, 12, 24, 48 and 72 h. XLZ7: *V. dahliae*-susceptible cotton vatiety; ZZM2: *V. dahliae*-resistant cotton vatiety. *GhUBQ7* is an internal reference gene. The data are three independent biological replicates, Significance analysis using T test (*P < 0.05; **P < 0.01).

qRT-PCR analysis showed that *GhWRKY53* was expressed in roots, stems and leaves of the ZZM2 and XLZ7, especially in the roots and stems. And its expression levels in leaves were relatively low. For XLZ7, a susceptible variety, *GhWRKY53* expression levels in the stems were significantly higher than roots. However, *GhWRKY53* expression levels were higher in the roots of disease-resistant variety ZZM2 (**Figure 2**). To further analyze the function of *GhWRKY53* in cotton verticillium wilt resistance, the two-leaf stage seeding was inoculated with *V. dahliae* by the root irrigation method (Xiong et al., 2019). The results showed that *GhWRKY53* expression levels were significantly up-regulated in the root and stem tissues of ZZM2 after 12 h of *V. dahliae* inoculated (**Figures 3A, B**). The *GhWRKY53* showed significantly up-regulated after 24 h of *V. dahliae* inoculated in the root and stem of XLZ7 (**Figures 3A, B**). However, the up-regulated of *GhWRKY53* expression levels of XLZ7 in roots and stems was not significant than ZZM2. In the resistant variety ZZM2, *GhWRKY53* had higher expression levels in the root and was more sensitive to the stress response of *V. dahliae*. It also indicated that *GhWRKY53* may play an important role in cotton resistance to verticillium wilt.

### 3.3 Expression pattern analysis of *GhWRKY53* under different hormone treatments

The upstream promoter sequence of *GhWRKY53* contains *cis*-acting elements of SA and MeJA, suggesting that *GhWRKY53* may be involved in disease resistance related to hormone response. Cotton seedlings treated with different hormones were sampled to detect the changes in *GhWRKY53* expression levels in the roots of the ZZM2 and XLZ7 after SA and MeJA treatment. The results show that the *GhWRKY53* expression levels were significantly up-regulated after 6, 12, and 24 h after SA treatment in ZZM2 (**Figure 3C**). However, the expression levels of *GhWRKY53* in sensitive variety XLZ7 showed a slow upward trend from 6 to 24 h after SA treatment (**Figure 3C**). After MeJA treatment, *GhWRKY53* expression levels in both XLZ7 and ZZM2 showed a trend of first downregulation and then upregulation, and *GhWRKY53* expression levels in both cotton varieties were significantly up-regulated at 48 h (**Figure 3D**). These results indicated that *GhWRKY53* was specifically regulated by SA and MeJA in different verticillium wilt resistant varieties.

### 3.4 Effect of silencing *GhWRKY53* on *V. dahliae* resistance of cotton

**Figure 4:**
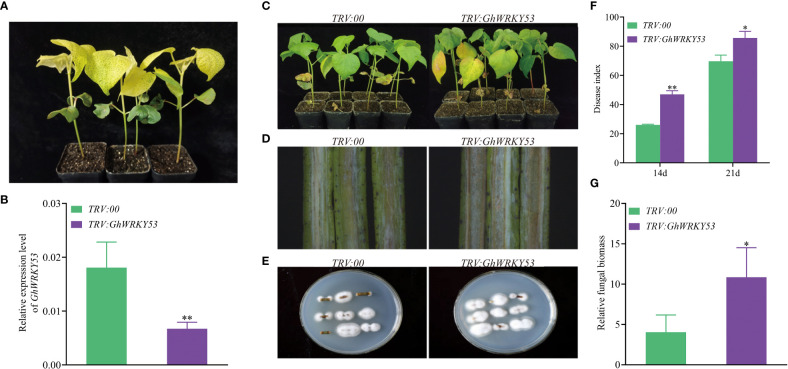
Silencing of *GhWRKY53* in *V. dahliae*- susceptible XLZ7. **(A)** Observation of the expected yellowing leaf phenotype in TRV::*GhCHLI* plants. **(B)** *GhWRKY53* expression in the TRV::00 and TRV::*GhWRKY53* plants. **(C)** Disease symptoms of the TRV::00 and TRV::*GhWRKY53* plants at 14 day post inoculation. **(D)** Comparison of vascular browning between TRV::00 and TRV::*GhWRKY53* plants at 14 day. **(E)** Fungal isolation in the stem sections from TRV::00 and TRV::*GhWRKY53* plants at 14 dpi. **(F)** Disease index for the TRV::00 and TRV::*GhWRKY53* plants at 14 and 21-day post-inoculating. **(G)** Quantification of the relative fungal biomass in stems of the TRV::00 and TRV::*GhWRKY53* plants at 21 day post inoculation. The data are three independent biological replicates, Significance analysis using T test (*P < 0.05; **P < 0.01).

**Figure 5:**
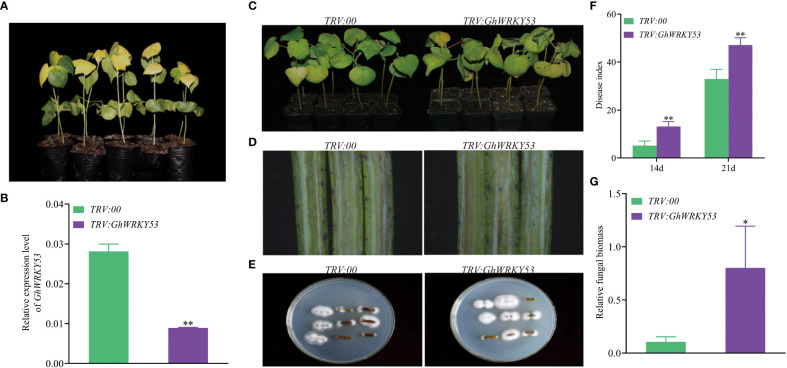
Silencing of *GhWRKY53* in *V. dahliae*- resistance ZZM2. **(A)** Observation of the expected yellowing leaf phenotype in TRV::*GhCHLI* plants. **(B)** *GhWRKY53* expression in the TRV::00 and TRV::*GhWRKY53* plants. **(C)** Disease symptoms of the TRV::00 and TRV::*GhWRKY53* plants at 14 day post inoculation. **(D)** Comparison of vascular browning between TRV::00 and TRV::*GhWRKY53* plants at 14-day. **(E)** Fungal isolation in the stem sections from TRV::00 and TRV::*GhWRKY53* plants at 14 dpi. **(F)** Disease index for the TRV::00 and TRV::*GhWRKY53* plants at 14 and 21-day post-inoculating. **(G)** Quantification of the relative fungal biomass in stems of the TRV::00 and TRV::*GhWRKY53* plants at 21-day post-inoculating. The data are three independent biological replicates, Significance analysis using T test (*P < 0.05; **P < 0.01).

To study the function of *GhWRKY53* on the cotton resistance to *V. dahliae*, VIGS was used to silence *GhWRKY53*. Seedlings infiltrated with *A. tumefaciens* carrying pTRV1/pTRV2::*GhCHLI* (plants-TRV:: *GhCHLI*) that targeted a gene encoding a ChlI subunit of magnesium chelatase were used as a positive control. After about ten days of injection, plants-TRV::*GhCHLI* showed a yellowing phenotype, indicating that VIGS system was functioning properly (**Figures 4A**, **5A**). Compared to the control plant (plants-TRV2::*00*), *GhWRKY53* expression levels in ZZM2 and XLZ7 respectively infiltrated with *A. tumefaciens* carrying pTRV1/pTRV2::*GhWRKY53*, decreased significantly, indicating that *GhWRKY53* was effectively silenced in XLZ7 and ZZM2 (**Figures 4B**, **5B**). And then cotton seedlings were inoculated with *V. dahliae* to verify plants resistance to *V. dahliae* after silencing *GhWRKY53*. At 14 days after inoculation with *V.dahliae*, *GhWRKY53*-silenced plants were more sensitive to *V. dahliae* infection than control plants in XLZ7. XLZ7-pTRV2::*GhWRKY53* also showed severe leaf yellowing and wilting, with more shedding of leaves (**Figure 4C**). The longitudinal section of stems after 21 d pathogen infection showed that the browning degree of the stems in the XLZ7-pTRV2::*GhWRKY53* was more significant (**Figure 4D**). Additionally, the pathogen colonies isolated from the stems of XLZ7-pTRV2::*GhWRKY53* were larger and darker than that of XLZ7 (**Figure 4E**). At 14 d and 21 d, the disease index of XLZ7-pTRV2::*GhWRKY53* plants were 47 ± 2.04 and 85.67 ± 3.70, respectively, significantly higher than that of TRV::00 plants at each time point (**Figure 4F**). Meanwhile, the relative contents of pathogen in the stem tissues and the recovery of pathogen were detected. The results showed that the biomass of pathogen in the stems of XLZ7-pTRV2::*GhWRKY53* was significantly higher than that of control plants, and its biomass was about three times that of XLZ7(**Figure 4G**). These demonstrated that silencing *GhWRKY53* further reduces the resistance of susceptible cotton variety XLZ7 to *V. dahliae*. In ZZM2, after 14 d inoculation, *GhWRKY53*-silenced plants (ZZM2-pTRV2::*GhWRKY53*) exhibited poor growth with severe disease and yellowing of leaves compared with control plants ZZM2. Compared with the control plant, leaves of ZZM2-pTRV2::*GhWRKY53* had significant withering (**Figure 5C**). The longitudinal section of stem showed that the accumulation of pathogen in ZZM2-pTRV2::*GhWRKY53* with severe browning was more than that in control plants, and the stem browning was severe (**Figure 5D**). The results of the pathogen recovery experiment showed that the colonies isolated from the stems of ZZM2-pTRV2::*GhWRKY53* were larger and had a denser villous shape than that of control plants (**Figure 5E**). The disease index of the ZZM2-pTRV2::*GhWRKY53* on the 14 d and 21d was 13.17 ± 2.02 and 47.17 ± 3.01 respectively, and far higher than the control plant(**Figure 5F**). Compared to the control plants, the stems of ZZM2-pTRV2::*GhWRKY53* accumulated more pathogen, and its biomass was about five times that of XLZ7 (**Figure 5G**). These were similar to the results observed in XLZ7, indicating that the silencing of *GhWRKY53* also significantly reduced the resistance of cotton to *V. dahliae*.

### 3.5 Effect of silencing *GhWRKY53* on SA and JA signaling pathways

**Figure 6:**
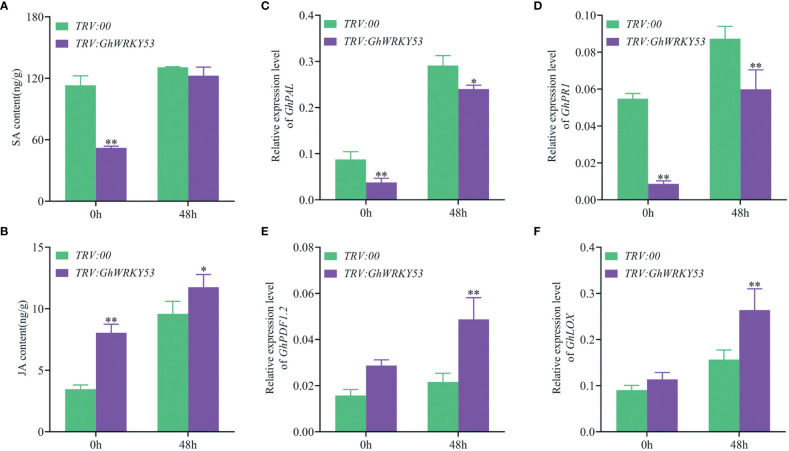
Changes in SA and JA content and the expression levels of related pathway genes in XLZ7 after *V. dahliae* inoculation. **(A)** Changes in SA content of TRV::00 and TRV::*GhWRKY53* plants after *V. dahliae* inoculation. **(B)** Changes in MEJA content of TRV::00 and TRV::*GhWRKY53* plants after *V. dahliae* inoculation. **(C)** qRT-PCR detects the expression changes of *GhPAL* after *V. dahliae* inoculation in TRV::00 and TRV::*GhWRKY53* plants. **(D)** *GhPR1*. **(E)** *GhPDF1.2*. **(F)** *GhLOX*. Significance analysis using T test (*P < 0.05; ** P< 0.01).

**Figure 7:**
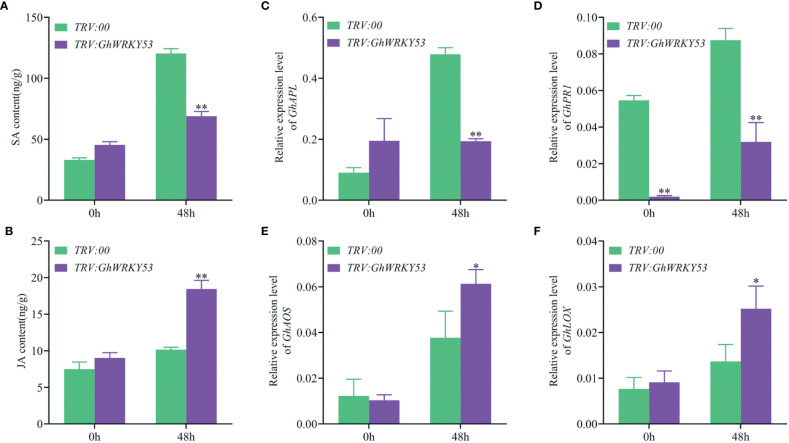
Changes in SA and JA content and the expression levels of related pathway genes in ZZM2 after *V. dahliae* inoculation. **(A)** Changes in SA content of TRV::00 and TRV::*GhWRKY53* plants after *V. dahliae* inoculation. **(B)** Changes in MEJA content of TRV::00 and TRV::*GhWRKY53* plants after *V. dahliae* inoculation. **(C)** qRT-PCR detects the expression changes of *GhPAL* after *V. dahliae* inoculation in TRV::00 and TRV::*GhWRKY53* plants. **(D)** *GhPR1*. **(E)** *GhPDF1.2*. **(F)** *GhLOX*. Significance analysis using T test (*P < 0.05; **P < 0.01).

To explore whether the deterioration of *V. dahliae* resistance in *GhWRKY53*-silenced plants is mediated by JA and SA signaling pathways, we measured JA and SA concentrations in the roots of the *GhWRKY53-*silenced plants and control plants, and the expression of signaling pathway-related genes also were analyzed. The results showed that SA content was up-regulated in both the control and silenced plants after inoculation with *V. dahliae* for 48 h in ZZM2 and XLZ7. For XLZ7, at 0 h after *V. dahliae* infected, the content of SA in *GhWRKY53*-silenced plants and control plants were 52.16 and 113 15 ng/g, respectively. And at 48 h, the content of SA increased to 122.51 and 130.67 ng/g, respectively (**Figure 6A**). Simultaneously, the content of JA in TRV::*GhWRKY53* and TRV::00 were 8.05 and 3.46 ng/g at o h, respectively. And increased to 11.75 and 9.59 ng/g at 48 h, respectively (**Figure 6B**). However, it was noted that the SA content in the *GhWRKY53*-silenced plants of XLZ7 was lower compared to the control plants at 0 h after *V. dahliae* inoculation (**Figure 6A**). Consistent with this result, SA biosynthesis gene (*GhPAL*) *and* response gene (*GhPRI*) were significantly up-regulated at 48 h (**Figures 6C, D**). And JA biosynthesis gene (*GhLOX*) and JA response gene (*GhPDF1.2*) also followed a similar trend (**Figures 6E, F**). For ZZM2, the SA content of TRV::*GhWRKY53* (68.92 ng/g) was significantly lower than TRV::00 (120.32 ng/g) at 48 h post-inoculation (hpi) (**Figure 7A**), with a similar trend being seen in the expression levels of SA biosynthesis gene (*GhPAL*) and response gene (*GhPRI*) (**Figures 7C, D**). The content of JA in TRV::*GhWRKY53* plants and *TRV::00* plants at 48 hpi increased 9.43 and 2.67 ng/g than 0 hpi after *V. dahliae* inoculation (**Figure 7B**), while the expression levels of JA biosynthesis genes (*GhAOS* and *GhLOX*) were also significantly induced at 48 hpi (**Figures 7E, F**). These results were support the notion that *GhWRKY53* plays a positive role in *V. dahliae* resistance by activating JA signaling pathway and repressing SA signaling pathway.

## 4 Discussion

As one of the most prominent transcription factor families in plants, members of the WRKY gene family play an essential role in many plant life processes, such as plant response to biotic and abiotic stresses, secondary metabolism of plants, and plant growth and development. Verticillium wilt caused by *Verticillium dahliae* is a destructive cotton disease causing severe yield and quality losses worldwide. WRKY transcription factors play important roles in plant defense against pathogen infection. However, little has been reported on the functions of WRKYs in cotton’s resistance to *V. dahliae*. Overexpression of *GhWRKY27a* reduced the tolerance of transgenic plants to drought stress and resistance to Rhizoctonia solani (Yan et al., 2020). In 2020, the reported that *GhWRKY70D1*3 negatively regulates cotton’s resistance to *V. dahliae* mainly through its effect on ET and JA biosynthesis and signaling pathways (Xiong et al., 2020). In this study, we explored the regulation of *GhWRKY53* in the *V. dahliae* resistance of cotton by silencing *GhWRKY53*. The gene-silenced plants had severe disease conditions and higher fungal contents, thereby indicating the positive regulatory role of *GhWRKY53* in the resistance mechanism of G. hirsutum to *V. dahliae*.

Studies have shown that WRKY TFs are involved in hormonal signal transduction processes (Zhang et al., 2015). At present, a large number of *cis*-acting elements related to SA and JA hormones have been identified in the promoter regions of WRKY TFs in Arabidopsis, tomato, wheat, and rice (Liu et al., 2007; Miao and Zentgraf, 2007; Murray et al., 2007; Van et al., 2014). In Arabidopsis, *AtWRKY18, AtWRKY40*, and *AtWRKY60* act as negative regulators of ABA signaling for seed germination and development (Chen et al., 2010). This study demonstrated multiple hormone-related *cis*-acting elements in the promoter regions of *WRKY53* genes in four cotton species by predicting the *cis*-acting elements of cotton *WRKY53*. Among them, the *cis*-acting elements of MeJA and SA were ubiquitously present in the promoter region of the *WRKY53* gene. In response to cotton hormone treatment, the results showed that *GhWRKY53* was induced by SA and MeJA at 24 and 48 h. Similar to the *WRKY53* gene in *G. barbadense*, *GbWRKY53* expression increased after SA and MeJA induction, with SA responding more compared to MeJA (Li et al., 2017). We speculated that *GhWRKY53* might act as a regulatory factor in the SA and MeJA signaling processes of cotton.

Researchers found that WRKY53 was specifically induced by SA following Arabidopsis infection to improve the plant’s defense against pathogenic fungus (Hu et al., 2012). In Arabidopsis, WRKY53 enhanced the disease resistance of plants by inhibiting the MeJA signaling pathway and activating the SA signaling pathway. In this study, both silenced and control plants were inoculated with *V. dahliae*, and the expression levels of SA and MEJA were measured. The results showed that the SA content in the silenced plants was significantly lower in different resistance varieties, while the MEJA content in the silenced plants was significantly higher at 48 h of inoculation. *GhPAL* and *GhPRI* have been confirmed to be involved in plant resistance to fungal infection and can be used as innate immunity markers in plants (Zhang et al., 2023). To further explore the function of *GhWRKY53* in defense against *V. dahliae*, the relative expression levels of the SA biosynthesis gene (*GhPAL*) *and* response gene (*GhPRI*) were monitored before and after *V. dahliae* treatment in gene-silenced plants. Additionally, the relative expression levels of the JA biosynthesis genes *GhLOX* and the JA signal response genes *GhPDF1.2* were monitored. Expression of the related pathway genes also followed a similar trend. The results suggest which further confirmed that *GhWRKY53* might act as a positive regulator in resistance to *V. dahliae*. It was speculated that the silencing of *GhWRKY53* inhibited the signaling pathway of SA and activated the signaling pathway of MEJA in cotton, thereby reducing the resistance of cotton to *V. dahliae*. These factors work together to enhance the resistance of cotton plants to *V. dahliae* infection. However, the interaction mechanism between MEJA and SA signaling pathways in cotton in response to *V. dahliae* requires further study.

## 5 Conclusion

In this study, We identified and obtained the gene encoding type III WRKY transcription factor *GhWRKY53* in upland cotton. Expression of *GhWRKY53* and the contents of SA and MeJA in different resistant varieties inoculated with *V. dahliae* showed that *GhWRKY53* mediated SA and MeJA signal transduction. Silencing of *GhWRKY53* inhibited the SA pathway and activated the MEJA pathway, thereby reducing the resistance of plants to *V. dahliae*. Our study demonstrated that *GhWRKY53* can change the tolerance of upland cotton to *V. dahliae* by regulating the expression of SA and MEJA pathway-related genes.

## Data availability statement

The original contributions presented in the study are included in the article/**Supplementary Material**. Further inquiries can be directed to the corresponding authors.

## Author contributions

YZ, YL, and FL designed and supervised the research. HC, FL, and YZ revised the manuscript. JS and YW guided the content of the article. YL, XZ, and JZ performed the data analysis. YL and HC finished the writing of the manuscript. All authors contributed to the article and approved the submitted version.
